# Progress and Prospects of Electrochemiluminescence Biosensors Based on Porous Nanomaterials

**DOI:** 10.3390/bios12070508

**Published:** 2022-07-11

**Authors:** Chenchen Li, Jinghui Yang, Rui Xu, Huan Wang, Yong Zhang, Qin Wei

**Affiliations:** 1 Collaborative Innovation Center for Green Chemical Manufacturing and Accurate Detection, Key Laboratory of Interfacial Reaction & Sensing Analysis in Universities of Shandong, School of Chemistry and Chemical Engineering, University of Jinan, Jinan 250022, China; lichenchen1107@126.com (C.L.); sdjndxwq@163.com (Q.W.); 2 Provincial Key Laboratory of Rural Energy Engineering in Yunnan, Yunnan Normal University, Kunming 650500, China; ynnu_yangjh2002@126.com (J.Y.); ecowatch_xr@163.com (R.X.)

**Keywords:** porous nanomaterials, electrochemiluminescence, metal-organic frameworks, covalent organic frameworks, metal-polydopamine frameworks, biosensors

## Abstract

Porous nanomaterials have attracted much attention in the field of electrochemiluminescence (ECL) analysis research because of their large specific surface area, high porosity, possession of multiple functional groups, and ease of modification. Porous nanomaterials can not only serve as good carriers for loading ECL luminophores to prepare nanomaterials with excellent luminescence properties, but they also have a good electrical conductivity to facilitate charge transfer and substance exchange between electrode surfaces and solutions. In particular, some porous nanomaterials with special functional groups or centered on metals even possess excellent catalytic properties that can enhance the ECL response of the system. ECL composites prepared based on porous nanomaterials have a wide range of applications in the field of ECL biosensors due to their extraordinary ECL response. In this paper, we reviewed recent research advances in various porous nanomaterials commonly used to fabricate ECL biosensors, such as ordered mesoporous silica (OMS), metal–organic frameworks (MOFs), covalent organic frameworks (COFs) and metal–polydopamine frameworks (MPFs). Their applications in the detection of heavy metal ions, small molecules, proteins and nucleic acids are also summarized. The challenges and prospects of constructing ECL biosensors based on porous nanomaterials are further discussed. We hope that this review will provide the reader with a comprehensive understanding of the development of porous nanomaterial-based ECL systems in analytical biosensors and materials science.

## 1. Introduction

Among various electroanalysis techniques, the electrochemiluminescence (ECL) method has attracted widespread attention due to its merits of high sensitivity and excellent analytical performance [1,2,3]. In the mid-1960s, Hercules, Santhanam, and Bard reported the first study on ECL [4,5]. Since then, the ECL technology has received wide attention from the scientific community. The ECL is a kind of new assay that combines two analytical methods: chemiluminescence (CL) and electrochemical techniques. Different from the traditional CL assay, ECL does not require an external excitation light source, so it has the advantages of a wider linear detection range, a better repeatability and anti-interference, a small background value, a good accuracy, and a high sensitivity [6,7]. The equipment and instruments for fabricating an ECL platform are usually small, and the preparation process is relatively simple and controllable [8,9]. Furthermore, ECL analysis can achieve continuous measurement, which is very popular and appropriate in the field of biochemical analysis, immunoassay, and pharmaceutical analysis. Attributing to these unique advantages, it has become one of the most highly interesting research areas for researchers in the field of analytical chemistry and been regarded as a very promising analytical assay [10,11,12].

With the inherent merits of a large specific surface area, a high porosity, an adjustable pore size and structure, and easy modification, porous nanomaterials have great potential in the fields of multiphase catalysis, gas adsorption and separation, drug transport, and biosensing [13,14,15,16]. In the field of ECL research, it is interesting that porous nanomaterials can not only be used as carriers for loaded luminophores, catalysts for accelerating the decomposition of catalytic co-reactants, and nanoreactors for accommodating ECL systems, but also accelerate the process of substance transport and charge transfer, all of which make ECL systems based on porous nanomaterials have a strong ECL response and a high sensitivity [17,18,19,20,21].

Sensitivity, stability, and reproducibility are important indicators for ECL biosensors. In order to improve the performance of ECL biosensors, it is particularly important to find and develop luminophores with strong ECL signals and a high stability. Conventional luminophores, such as g-C_3_N_4_, have an excellent ECL response, but their sheet-like structure makes their specific surface area relatively small, which reduces the probability of contact between the luminophore and the co-reactant and seriously affects their ECL luminescence efficiency. Luminol, tris-2-2′-bipyridyl ruthenium (i.e., Ru(bpy)_3_^2+^), and their derivatives possess good water solubility, making it difficult to apply them alone as luminophores in aqueous solutions. Therefore, finding nanomaterials with a large specific surface area and a high porosity as carriers of ECL luminophores or preparing ECL materials with a large specific surface area and a high porosity is of extraordinary significance for the preparation of high-performance ECL biosensors. In recent years, porous nanomaterials, such as ordered mesoporous silica (OMS) [22], metal–organic frameworks (MOFs) [23], covalent organic frameworks (COFs) [24], and metal–polydopamine frameworks (MPFs) [25] have received extensive attention in the study of ECL biosensors because of their large specific surface area, high porosity, tunable pore size and structure, and easy modification. It has also demonstrated excellent performance in the detection of heavy metal ions, small molecules, proteins, and nucleic acids.

**Figure 1:**
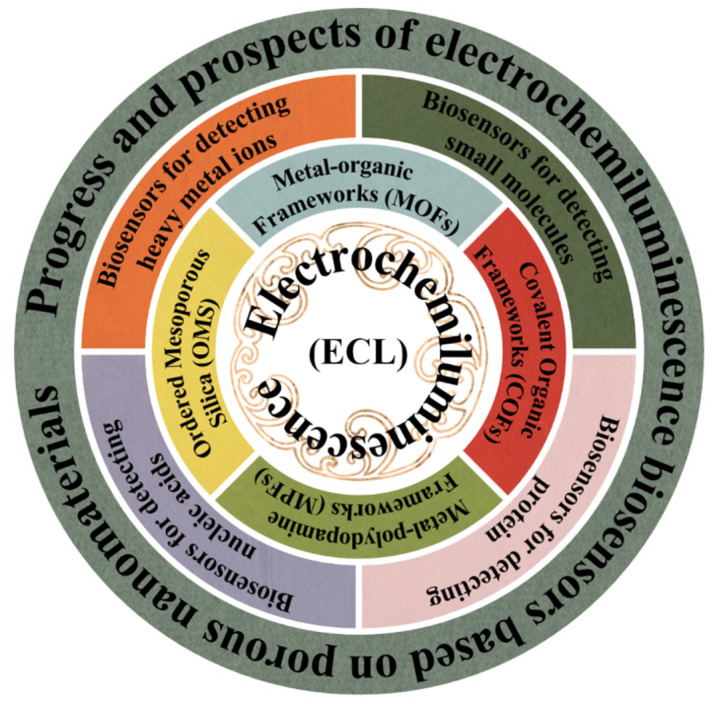
Schematics illustrating the application of ECL biosensors based on porous nanomaterials, as reviewed in this paper.

In this review, we present a detailed description of porous nanomaterial-based ECL biosensors, combining the basic construction process and the applied reaction mechanism to show the innovative applications of porous nanomaterials in ECL biosensors, as shown in Figure 1. We further discuss the challenges and the prospects of ECL systems based on porous nanomaterials. This review will enable readers to understand the relevant contents comprehensively and find more innovative applications.

## 2. Synthesis of Porous Nanomaterials with ECL Properties

Numerous experimental results have shown that ECL biosensors based on porous nanomaterials can effectively enhance the ECL performance and enhance the accuracy of analysis [26,27]. Therefore, we firstly summarize and discuss the preparation of those porous nanomaterials with ECL properties, such as OMS, MOFs, COFs, and MPFs.

### 2.1. Ordered Mesoporous Silica (OMS) with ECL Properties

OMS has a wide range of applications in ECL biosensors, because of its excellent morphological characteristics, excellent stability, and simple preparation method. It is usually used as a carrier to load ECL substances by means of doping or coating techniques [28,29]. To make the discussion clearly, Table 1 summarizes several kinds of OMS-based nanocomposites being applied in the field of ECL biosensors and the corresponding synthesis strategies in recent years.

As shown in Table 1, the methods for the synthesis of OMS with ECL properties are broadly divided into two categories. One is to synthesize silica nanoparticles (SiO_2_ NPs) and obtain OMS-based nanocomposites through sodium hydroxide (NaOH) etching on this basis. The other is to encapsulate small organic particles in SiO_2_ NPs by the microemulsion method and then obtain OMS-based nanocomposites by the high temperature calcination method [41]. The difficulty in controlling the process of etching SiO_2_ NPs by NaOH makes it hard to get OMS with uniform pore channels by this etching method. Recently, the OMS prepared through the microemulsion method can effectively solve such problems [30,31]. For example, Lin et al. prepared OMS using this method, in which the luminescent g-C_3_N_4_ was combined with the previously prepared OMS by post-modification method as an efficient ECL probe. Based on this, the prepared sensor showed excellent correlation in the range of 0.1 nm–10 μm with an extremely low limit of detection (LOD) of 33 pM.

In another work, You et al. directly employed a microemulsion method to encapsulate Ru(bpy)_3_^2+^ and CN QDs in SiO_2_ NPs. The electrons were transferred from CN QDs to Ru(bpy)_3_^2+^ through an intramolecular pathway, which shortened the distance between the electron transfer and thus improved the luminescence efficiency, yielding a self-enhanced ECL signal probe [32]. In a creative study, Jin et al. prepared a homogeneous Ru@SiO_2_ NP colloidal solution and then applied it to develop Ru@SiO_2_ NP nanomembranes on the surface of indium tin oxide glass (ITO) by a liquid–liquid interface self-assembly method. The obtained Ru@SiO_2_ NP nanomembrane can be used as both an enhanced substrate and a luminol enricher. A self-enhanced ECL biosensor was constructed based on the intense luminescence of the Ru@SiO_2_ NP nanomembrane and the enrichment of Ru(bpy)_3_^2+^ molecules on the surface of the Ru@SiO_2_ NP nanomembrane [33]. In order to study the effect of the preparation process of nanocomposites on the properties of ECL, Shen et al. prepared CdTe@SiO_2_ and SiO_2_@CdTe NPs via microemulsion and post-modification methods, respectively. Interestingly, CdTe@SiO_2_ with ordered mesopores is more efficient and less bio toxic for the preparation of ECL biosensors. The ECL immunosensor for the detection of methemoglobin was prepared using CdTe@SiO_2_ as the signal probe with a good linearity in the range of 1.0 pg/mL to 100 ng/mL and the LOD was 0.22 pg/mL [34].

### 2.2. Metal–Organic Frameworks (MOFs) with ECL Properties

MOFs are a new type of porous material formed by organic ligands and metal ions or metal clusters linked by coordination bonds. Due to it inherit merits, such as a large surface area, a high porosity, abundant active sites, and a strong mass transfer capability, it has excellent performance in the field of novel materials and has a wider application in the fields of multiphase catalysis, gas adsorption and separation, drug transport, and biosensing [42,43,44,45]. Table 2 summarizes recent reported MOFs used in the field of ECL biosensors and the synthesis strategies of their composite, especially illustrating post-synthetic modifications, in situ synthesis and self-luminescent MOFs.

#### 2.2.1. In Situ Synthesis

Although MOFs have a high porosity and a tunable pore size, the pore size could be fixed along with the successful preparation of MOFs. Most classical MOFs contain only micropores, which leads to the inability of guest luminophores to easily enter the MOF interior through the pores, making the luminophore loading capacity of MOF materials greatly be reduced [75]. To solve this problem, Yang et al. wrapped the luminescence inside the MOF material by an in situ synthesis method during the MOF growth process, which resulted in a greatly enhanced loading capacity of the guest luminescence, and thus prepared the nanocomposite with a high-intensity ECL response [47].

**Figure 2:**
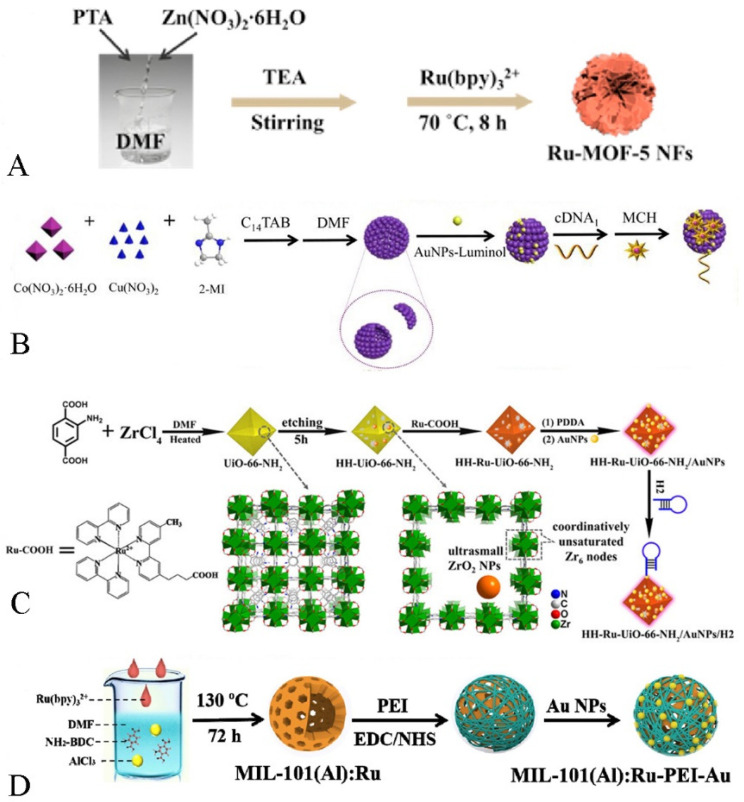
(**A**) The preparation of Ru-MOF-5 NFs [53]. Copyright © 2021, Elsevier. (**B**) The synthesis process of Cu/Co-MOF-luminol-AuNPs [54]. Copyright © 2021, Elsevier. (**C**) Preparation of HH-Ru-UiO-66-NH_2_/Au NPs/H_2_ [55]. Copyright © 2021, American Chemical Society. (**D**) The synthesis steps of MIL-101(Al): RuPEI-Au [46]. Copyright © 2019, Elsevier.

As we all know, as an excellent luminescent substance, Ru(bpy)_3_^2+^ is often used in the process of constructing various ECL biosensors. For instance, Cao et al. encapsulated Ru(Bpy)_3_^2+^ molecules into NH_2_-UiO-66 through the ligand effect during the growth of NH_2_-UiO-66. The open channels or active cavities of MOFs could not only maintain the excellent ECL response of Ru(bpy)_3_^2+^, but also enrich the co-reactants, enabling the ECL biosensor to exhibit a highly selective and efficient ECL response, thus facilitating the ECL biosensor for ultra-sensitive and accurate analyses of the β-amyloid [48]. As shown in Figure 2D, Wang et al. applied mesoporous, hollow MIL-101(Al)-NH_2_ in an ECL system by the in situ growth process and achieved a large and stable loading of Ru(bpy)_3_^2+^. Additionally, the authors also made poly(ethylenediamine) as a co-reactant and combined it with MIL-101(Al)-NH_2_ through covalent bonding, which not only prevented the leakage of Ru(bpy)_3_^2+^, but also made the Ru complex produce strong and stable ECL signals through self-enhancement effect [46].

In addition to Ru(bpy)_3_^2+^, quantum dots (QDs) are often used in the field of ECL biosensors as an efficient and stable luminescent [76,77]. In the recent study, Tan et al. attached a large number of CdS QDs to chain-like polyethylenimine (PEI) via amide bonding, wrapped the modified PEI on the surface of MIL-53(Al), achieved massive loading of CdS QDs, and finally prepared MOF-based ECL signaling probes [78]. In another study, Deng et al. successfully encapsulated ZnSe QDs in Fe (III)-MIL-88B-NH_2_ through the in situ growth process. Fe(III)-MIL-88B-NH_2_ can not only achieve massive loading of ZnSe QDs, but it also contains amino groups for catalyzing the conversion of the co-reactant S_2_O_8_^2−^ into sulfate anions (SO_4_^•−^), which shortens the electron transfer distance and reduces the energy loss, enabling the ECL property of ZnSe QDs [49]. Although all of the above works demonstrate that the preparation of nanomaterials with ECL properties by the in situ growth method is an excellent strategy, the leakage of luminescent material is still an inevitable issue in the practical operations.

#### 2.2.2. Post-Synthesis Modification

Post-synthesis modification is an effective means to prepare MOFs with ECL properties. The mechanism is mainly to attach luminophores to the surface of MOFs by ligand reaction, electrostatic force adsorption, or amide bonding, so that the MOFs materials, originally without ECL properties, become nanocomposites with ECL properties [79,80].

For example, As shown in Figure 2A, Wei et al. indicated that a flower-like nanomaterial with ECL properties was successfully prepared by loading Ru(bpy)_3_^2+^ onto the surface of MOF-5 NFs via electrostatic force adsorption, exhibiting an excellent ECL response [53].

It is worth noting that MOFs with catalytic properties act as carriers, not only to enrich the co-reactants but also to catalyze the co-reactants so that the ECL response is enhanced. As shown in Figure 2B, Zhou et al. successfully prepared nanocomposites with ECL properties by ligand bonding luminol to the MOF. The obtained hollow Cu/Co-MOF not only acted as a carrier but also could catalyze the generation of more O^2−^ from H_2_O_2_, which greatly enhanced the ECL response by means of this material [54].

As shown in Figure 2C, Yuan et al. connected the luminescent material (Ru(Bpy)_2_(Mcpbpy)^2+^) with the carrier MOF (HH-UiO-66-NH_2_) through amide bonding. On the one hand, the hierarchical pore shell and hollow cavity of HH-UiO-66-NH_2_ exposed more amino groups, which made the loading of the luminescent greatly increased. On the other hand, the HH-UiO-66-NH_2_ surface amino group could catalyze the generation of SO_4_^•^^−^ from S_2_O_8_^2−^, which greatly shortened the distance between the co-reactant and the luminophore, making the charge transfer more efficient and thus enhancing the ECL signals [55].

#### 2.2.3. Self-Luminous MOFs

Luminous metal-organic frameworks (LMOFs) that consist of organic bridging ligands and metal-linked nodes are novel porous nanomaterials commonly used in ECL biosensors in recent years [81]. Since LMOFs have multiple structural units, the ECL response may come from metal centers and ligands within the MOF, and the optical properties can be modulated by the interactions between the building components. Based on this, the problems of low loading of modified luminescent substances and leakage from the in situ-grown luminescent substances after MOF synthesis might be effectively solved [62,82].

**Figure 3:**
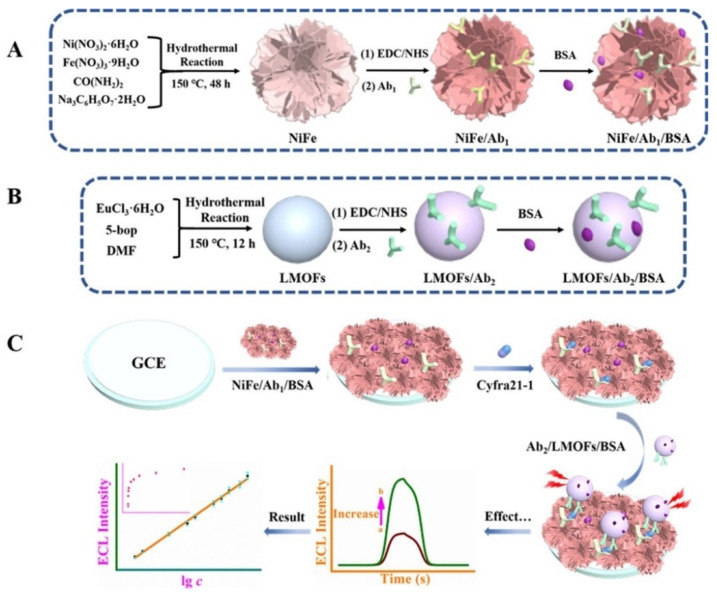
Preparation of NiFe Complex/Ab_1_/BSA Bioconjugate (**A**), Ab_2_/LMOFs/BSA Bioconjugate (**B**), and Formation Route of the Suggested Signal-Enhanced ECL Model (**C**) [61]. Copyright © 2021, American Chemical Society.

Lanthanide rare-earth metal ions are an important emerging LMOFs precursor because of their unique [Xe]4f^n^ (n = 0–14) ground-state electronic grouping pattern, which is prone to 4f–4f transitions and possesses abundant ladder electron energy levels and sharp emission bands. As shown in Figure 3, Wei et al. prepared self-luminous (Ln) metal-organic frameworks (Ln-MOFs) by hydrothermal treatment using Eu (III) ions and 5-boryl-isophthalic acid (5-bop) as precursors. The 5-bop in the triplet excited state can transfer its own energy to the Eu(III) ion by emitting ultraviolet light. When the Eu(III) ion gains energy from the ligand, it can jump to a higher energy level and release more light energy when it falls back to the ground state. The more intense ECL signal is obtained through the antenna effect of Eu(III) ions. The prepared sensor showed a good linearity in the range of 0.005 to 100 ng/mL, and the obtained LOD was only 0.126 pg/mL [61].

Ru(bpy)_3_^2+^ and its derivatives can not only prepare porous nanomaterials with ECL properties by post-synthesis modification or in situ growth, but also act as ligands for the direct synthesis of self-luminous MOFs involved in the construction of ECL biosensors [73,83].

In a pioneering work conducted by Yan et al., self-luminous Ru-MOF has been synthesized by using the autoloading Ru(dcbpy)_3_^2+^ and zinc ions as precursors. The obtained Ru-MOF nanosheets expose more active centers, promote closer contact with the target molecule, and have shorter diffusion distances for ions, electrons, and co-reactants. The excellent property makes the self-luminous Ru-MOF show great potential as a new Faraday cage for developing a biosensing platform [62].

**Figure 4:**
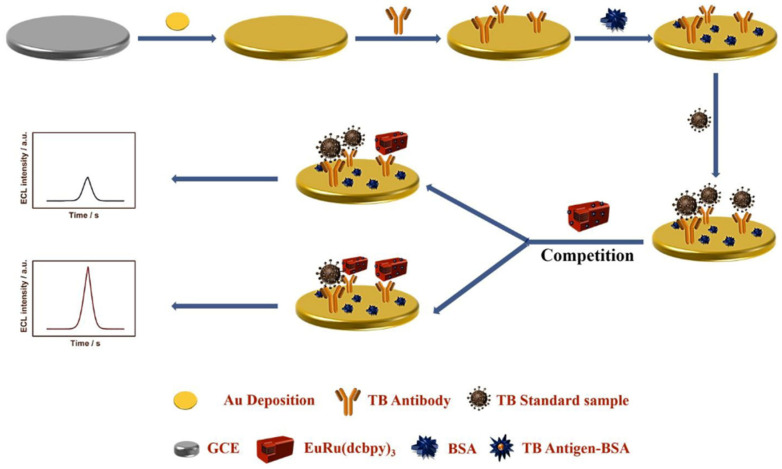
Annihilation luminescent Eu-MOF as a near-infrared electrochemiluminescence probe for trace detection of trenbolone [63]. Copyright © 2022, Elsevier.

As shown in Figure 4, in another interesting work, Zhao et al. synthesized a new type of Eu-MOF by a hydrothermal method using Eu (III) ions and Ru(dcbpy)_3_^2+^ as precursors. The Eu-MOF can undergo redox reactions and energy transfer between its ligand molecules and achieve annihilation luminescence without any additional co-reactants. At the same time, the antenna effect of Eu (III) ions in Eu-MOF is generated. In other words, when Eu (III) ions absorb the energy from the ligand, the luminescence efficiency is greatly increased, and the secondary near-infrared (NIR-II) luminescence is obtained. The prepared ECL sensor using Eu_2_[Ru(Dcbpy)_3_]_3_ as the ECL signal probe was extremely resistant to interference and achieved the rapid and sensitive detection of trenbolone in the range of 5 fg/Ml–100 ng/Ml with a lower LOD of 4.83 fg/Ml [63].

Although Ru(bpy)_3_^2+^ and its derivatives have very common applications in the field of ECL, their high price and the biotoxicity carried by the co-reactants limit the current application of Ru-containing MOFs.

**Figure 5:**
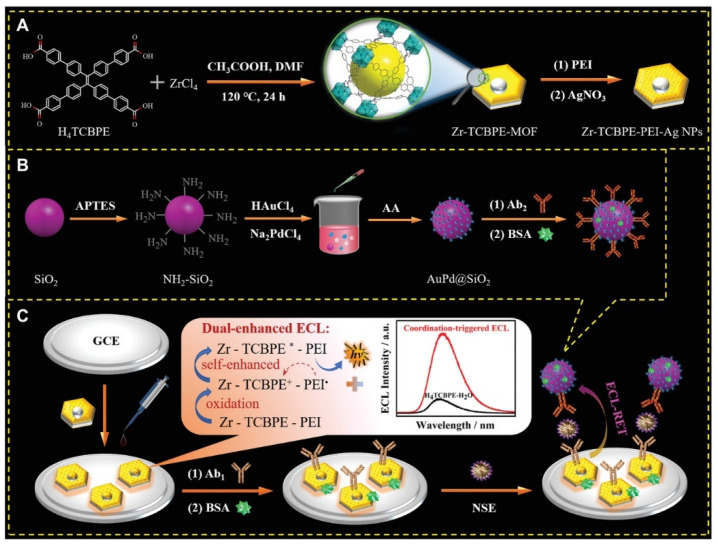
Schematic illustration for the synthesis of (**A**) Zr-TCBPE-PEI-Ag NPs substrate and (**B**) Ab_2_-AuPd@SiO_2_ bioconjugate, and (**C**) the fabrication process of the proposed ECL immunosensor with possible ECL-enhancing effects and luminescence mechanism [64]. Copyright © 2022, John Wiley and Sons.

In particular, aggregation-induced ECL (AI-ECL) was firstly discovered by Luisa De Cola’s team in 2017 [84]. With the continuous development in recent years, some excellent AI-ECL materials have emerged. Recently, researchers found that tetraphenylethylene (TPE) and its derivatives have the characteristics of a high ECL efficiency via easy modification. Compared with its aggregates and monomers, MOFs prepared based on TPE showed a stronger ECL response. For instance, Yuan et al. successfully prepared a novel 2D ultrathin MOF material based on the aggregation-induced emission (AIE) ligand H4ETTC and used it to construct a novel ECL biosensor for the ultrasensitive detection of CEA. The newly synthesized AIE luminogen (AIEgen)-based MOF (Hf-ETTC-MOF) yielded a higher ECL intensity and efficiency than H_4_ETTC monomers, H_4_ETTC aggregates and 3D bulk Hf-ETTC-MOF did [85]. As shown in Figure 5, Wei et al. synthesized a dumbbell-shaped metal–organic backbone with high luminescence efficiency by combining the aggregation-induced luminescent material H_4_TCBPE with Zr(IV) ions. The obtained Zr-TCBPE-MOF possesses a more excellent ECL performance compared to the monomer and aggregates of H_4_TCBPE. In addition, the authors combined Zr-TCBPE-MOF with polyethyleneimine (PEI) to prepare a unique self-reinforced Zr-TCBPE-PEI electroluminescent complex, which could effectively avoid the bio-toxicity of the co-reactant and exhibit a more dramatic ECL response. The prepared ECL sensor showed good correlation in the range of 0.0001–10 ng/mL and the lower LOD was 52 fg/mL, providing an effective way for the early and sensitive detection of small cell lung cancer [64].

### 2.3. Covalent Organic Frameworks (COFs) with ECL Properties

COFs is a class of organic porous crystalline materials composed of light elements (C, O, N, B, etc.), which are connected to each other by covalent bonds. Due to their structural designability, low density, high specific surface area, easy modification, and functionalization, COFs have been widely investigated and shown excellent prospects for applications in the fields of gas storage and separation, non-homogeneous catalysis, energy storage materials, optoelectronics, sensing, and drug delivery [86].

**Figure 6:**
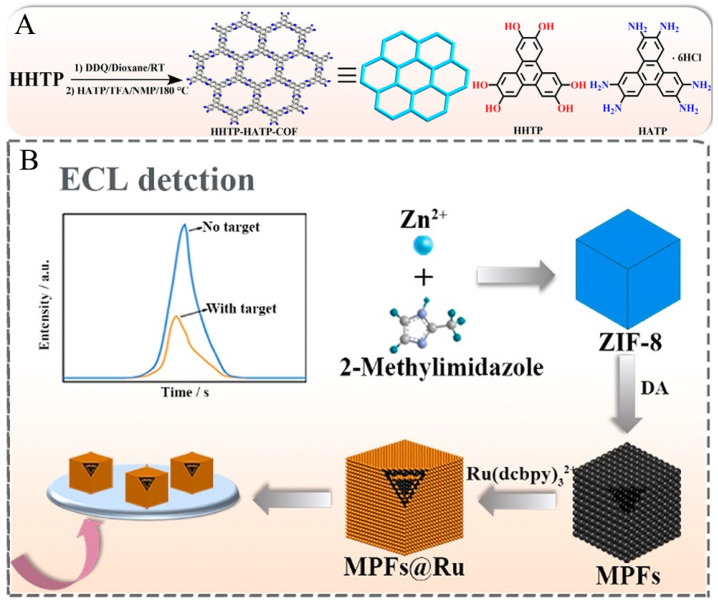
(**A**) Synthesis of HHTP-HATP-COF [88]. Copyright © 2022 American Chemical Society. (**B**) Preparation of MPFs@Ru [25]. Copyright © 2021 Elsevier.

Although COFs materials currently synthesized by solvothermal synthesis and Knoevenagel polycondensation reaction are often used for ECL biosensors, there are not so many articles reported about the application of COFs in the development of ECL biosensors. Recently, Zhuo et al. prepared a nanocomposite with ECL properties by attaching Ru(bpy)_3_^2+^ to the surface of COF-LZU1. Since COF-LZU1 has a hydrophobic porous structure and TPrA is lipophilic, a large amount of TPrA in aqueous solution can be enriched into the hydrophobic inner cavity of COF-LZU1, which will shorten the distance between the luminescent material and the co-reactant and increase the concentration of co-reactant around the luminescent, resulting in a greatly enhanced ECL response [87]. However, the conductivity of COFs materials limits its application in the ECL field, which may be one of the reasons for limiting applications of COFs in the ECL biosensing study. To solve this problem, Yuan et al. provided a novel strategy. They prepared a conductive COF (HHTP-HATPCOF), as shown in Figure 6. In their work, since HHTP-HATP-COF has a large amount of ECL-emitting material and its conductive porous backbone accelerates the charge transfer in the whole backbone, the ECL response of this composite is greatly enhanced [88].

### 2.4. Metal–Polydopamine Frameworks (MPFs) with ECL Properties

MPFs are a new hybrid material that perfectly combine the advantages of both MOFs and polydopamine (PDA) [89]. The large specific surface area and high porosity can be used to obtain a strong ECL signal by increasing the loading of luminophores. The PDA structure contains active double bonds that can chemically react with multiple groups to connect luminophores. The PDA structural fragment is a conjugated system that can generate π–π stacking with luminophores containing π bonds and thus adsorb luminophores [90,91].

As shown in Figure 6, Ma et al. prepared the MOF (ZIF-8) as the basic framework by the self-assembly method at first. After that, the hollow and porous metal–polydopamine frameworks (MPFs) were gradually formed by reacting with dopamine in a mixture of ethanol and Tris-HCl buffer, in which the polydopamine continuously replaced the original ligands through coordination reactions. Finally, the nanocomposites with ECL properties were formed by adsorption of Ru(bpy)_3_^2+^ through π–π stacking [25]. Although MPFs have many advantages, there are still less related studies on applying MPFs in preparing ECL biosensors. So, it is hoped that MPFs can achieve greater breakthroughs in the field of ECL biosensors through the continuous efforts of researchers.

## 3. Application of ECL Biosensors Based on Porous Nanomaterials

Due to their excellent stability and selectivity, porous nanomaterials have a wide range of applications in the field of ECL biosensors [92,93]. Here, we focus on introducing the applications of ECL biosensors based on porous nanomaterials in the detection of heavy metal ions, small molecules, proteins, and nucleic acids in recent years.

### 3.1. Biosensors for Detecting Heavy Metal Ions

It is well known that heavy metal ions have a great impact on human health and the natural environment, especially the intake of large amounts of heavy metal ions can cause irreversible damage to the human body, so the reliable and accurate detection of heavy metal ions is of great importance [94]. For example, You et al. prepared a label-free ECL biosensor for the detection of Hg^2+^ based on the different affinity of Ru-QDs@SiO_2_ nanocomposites for loading single-stranded DNA (SsDNA) and Hg^2+^-initiated double-stranded DNA (DsDNA). When no ions of Hg^2+^ are present, single-stranded DNA is attached to the Ru-QDs@SiO_2_ surface by hydrogen bonding, i.e., electrostatic force adsorption, which leads to the quenching of the ECL signal. When Hg^2+^ is present, part of the single-stranded DNA on the surface of Ru-QDs@SiO_2_ is guided to form a stable dsDNA, allowing part of Ru-QDs@SiO_2_ to exist in a free state, reducing the quenching of the ECL signal to single-stranded DNA [32]. Additionally, You et al. attached one end of the Hg^2+^ aptamer to NH_2_-Ru@SiO_2_-NGQds through an amide bond and the other end to AuNPs on the surface of the glassy carbon electrode (GCE) through an Au–S bond. When Hg^2+^ is absent, the aptamer is a long chain, and there is a large spatial site resistance between the luminescent material and the electrode surface, and, thus, the ECL response is not strong. When Hg^2+^ is present, the aptamer bends due to the formation of a thymine-Hg^2+^-thymine (T-Hg^2+^-T) specific structure, which draws the distance between the luminescent material and the electrode surface and reduces the spatial potential resistance, making the ECL response greatly enhanced [35].

### 3.2. Biosensors for Detecting Small Molecules

Table 3 summarizes ECL biosensors for detecting the small molecule based on porous nanocomposites in recent years.

Chloramphenicol (CAP) is a broad-spectrum antibiotic that can effectively treat a variety of microbial infections such as typhoid fever, meningitis, and salmonellosis. Since the middle of last century, it has become a widely used antibiotic because of its low production cost and good drug stability. However, many studies in recent years have shown that excessive intake of CAP can inhibit bone marrow hematopoiesis, which in turn can severely damage the human hematopoietic system. Therefore, it is necessary to establish a rapid and sensitive detection method to accurately monitor CAP residues in the aqueous environment. Chen et al. synthesized black phosphorus QDs (BPQDs) into PTC-NH_2_ solution to synthesize nanocomposites with ECL properties (BP/PTC-NH_2_). An efficient and sensitive ECL sensor was prepared to detect CAP by combining BP/PTC-NH_2_ with Co-Ni/MOF as an ECL emitter via electrostatic adsorption. The composite material of co-Ni/MOF has a good catalytic effect and can catalyze the co-reactant K_2_S_2_O_8_ to generate more SO_4_^•−^, which enhances the ECL response of the system. When CAP is present, the specific recognition of the aptamer makes the aptamer detach from the surface of the luminescent material, which reduces the burst of the aptamer for the ECL response and thus enhances the ECL signal [56].

**Figure 7:**
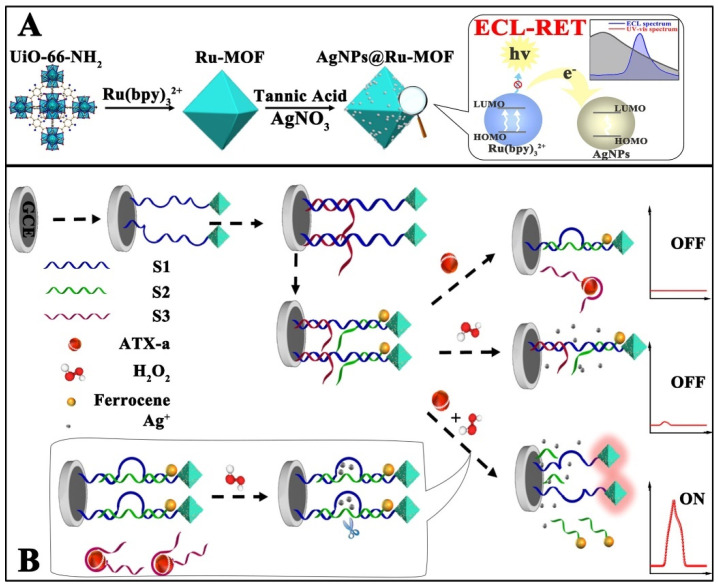
Schematic illustration of in situ generation of AgNPs@Ru-MOF and progress of ECL-RET (**A**), and the response process of ECL-RET aptasensor for ATX-a (**B**) [57]. Copyright © 2021, American Chemical Society.

Anatoxin-a (ATX-a) is a highly toxic alkaloid neurotoxin isolated from Anabaena flos-aquae (e.g., a semi-lethal dose of 200 μg/mL for rats) with a strong nicotine-like neuromuscular depolarization blocking effect, and animals poisoned will experience myofascicular twitching, corneal inversions, respiratory muscle spasms, and the animals will show symptoms such as muscle bundle convulsion, corkscrew, respiratory muscle spasm, and salivation after poisoning. Therefore, it is of great interest to find a rapid and sensitive test for the detection of ATX-a in water. As shown in Figure 7, Wang et al. proposed an ECL biosensor based on the ECL-RET strategy with a low background signal by means of double burst and dual stimulus response. The prepared ECL biosensor provides an accurate signal output for the ultrasensitive detection of ATX-a. Specifically, the authors wrapped Ru(bpy)_3_^2+^ in UiO-66-NH_2_ by the in situ growth method to act as an ECL signal probe, wrapped it with silver nanoparticle (AgNPs) shells as the main bursting agent, and tightly bound it to DNA-ferrocene (Fc). The AgNPs play an important role in the whole system, not only to close the permanent pore of UiO-66-NH_2_ and prevent the leakage of Ru(bpy)_3_^2+^, but also to act as a quencher to quench the ECL signal of Ru(bpy)_3_^2+^. Not only that, the AgNPs generated in situ can specifically recognize and break the substrate chain, generating an “On” signal, which helps to avoid false positive results. Thus, an ultra-sensitive detection of ATX-a was achieved in the range of 0.001 to 1 mg/mL, and the LOD was estimated to be 0.00034 mg/mL [57].

### 3.3. Biosensors for Detecting Protein

Cancer has now become a global difficult-to-treat disease, and it is well known that the treatment of early-stage cancer patients saves much more labor, money, and time than that of late-stage cancer patients, so early diagnosis of cancer is of great significance. The ECL analysis method provide a potential assay for the sensitive and selective detection of certain cancer disease-related biomarkers by coupling antigen–antibody specific binding with ECL technology. As shown in Table 4, we summarize some of the porous nanomaterial-based ECL sensors used for protein detection in recent years.

Mucin-1 (MUC1) is an important transmembrane glycoprotein that is considered an important biomarker for colon, breast, ovarian, and lung cancers. Recently, Yuan et al. prepared a novel multivacancy nanocomposite (Hf-TCBPE) with ECL properties based on the principle of matrix coordination-induced ECL (MCI-ECL) enhancement. The MOF constructed by Hf ions and TCBPE ligands has its internal spatial structure fixed, which restricts the intramolecular free motion of TCBPE and suppresses the nonradiative relaxation, and the high porosity of Hf-TCBPE enables both internal and external excitation of TCBPE, which greatly enhances its ECL response. An ECL biosensor for the detection of mucin 1 (MUC 1) was constructed by combining HF-TCBPE with a phosphate-terminated ferrocene (FC)-labeled hairpin DNA aptamer (FC-HP3) as a signal probe (HF-TCBPE/FC-HP3) with the aid of an exonuclease III (Exo III) cyclic amplification strategy [65]. Another novel piece of research is that Xiao et al. discovered that the ECL of the material could be enhanced by restricting intramolecular motion, and based on this principle, a two-dimensional ultrathin MOF (Zr_12_-ABD) with AI-ECL properties was prepared. In 2D MOF, the ligand 9,10-anthracene dibenzoate is immobilized, which restricts its intramolecular motion and suppresses the energy loss due to spin, allowing more energy to be released in the form of light energy and significantly enhancing the ECL response. Meanwhile, the ultrathin multivacancy structure of 2D MOF not only allows more co-reactants to enter the interior of MOF, but also reduces the migration distance between the electrons, ions, and co-reactants due to the smaller spatial site resistance, which reduces the energy loss and further enhances the ECL response of Zr_12_-ABD. A biosensor for the sensitive detection of mucin 1 was prepared by combining Zr_12_-ABD nanomaterials with a bipedal walking molecular machine. The ECL signal decreased with an increasing concentration of MUC1 in the range of 1 fg/mL to 100 ng/mL, showing a good linearity, and the LOD of the prepared ECL sensor was only 0.25 fg/mL [66].

CYFRA21-1 is considered to be a tumor marker mainly used for the detection of lung cancer and is especially valuable for the diagnosis of non-small cell lung cancer (NSCLC). In a study, Wei et al. creatively prepared a rare earth (Ln) metal–organic backbone (LMOF) with ECL properties. Ln-MOF was prepared from a precursor containing Eu(III) ions and 5-boronic acid isophthalic acid (5-bop). The ligand 5-bop produces a triplet state upon UV excitation, which triggers the red light emission of Eu(III) ions and enhances the ECL response. The electron-deficient boric acid reduces the energy transfer efficiency from the triplet state of 5-bop to the Eu(III) ion, resulting in both being efficiently excited under a single excitation. In addition, the synthesized flower-like Ni/Fe composites (Ni/Fe 1:1) have more active centers, higher stability, and good electrical conductivity by gradually adjusting the atomic ratio of Ni/Fe. An ECL immunosensor for the highly sensitive detection of CYFRA21-1 was prepared using Ln-MOF as the ECL emitter and flower-like Ni/Fe composite as the substrate, and the prepared Eu-LMOF showed good performance characteristics in the ECL immunoassay with the LOD of 0.126 pg/mL [61].

Ju et al. synthesized a MOF with ECL properties (Tb-Cu-PA MOF) using luminescent Tb^3+^ and catalytic Cu^2+^ ions as metal linkers and isophthalic acid (PA) as a bridging ligand. The doping of Cu^2+^ significantly reduced the size of the MOF and produced a strong and stable ECL signal. Therefore, the authors prepared a novel ECL immunosensor for the sensitive detection of CYFRA21-1 by using the synthesized Tb-Cu-PA MOF as an ECL emitter and Ni–Co layered double hydroxide (LDH) containing ZIF-67 nanocubes as a substrate. Compared with ZIF-67, ZIF-67@LDH has larger specific surface area and more active centers. After depositing palladium nanoparticles (Pt NPs) on ZIF-67@LDH nanocubes, it can improve the charge transport and electrocatalytic performance, catalyze S_2_O_8_^2−^ to produce more SO4^•−^, and obtain more intense ECL signals. The linear range of the successfully prepared ECL immunosensor was 0.01–100 ng/mL with a LOD of 2.6 pg/mL [67].

Except for the biomarkers, biological enzymes also play an irreplaceable role in human life activities. The abnormal activity of certain enzymes can lead to disorders in human functions. Therefore, to develop a rapid and highly sensitive measurement of some enzymes is important for clinical diagnosis and basic biochemical research. The sensitive detection of thrombin (TB), an important biomarker that plays an important role in hemostasis and thrombosis, has attracted great interest. In a study, Yuan et al. prepared a hollow graded MOF (HH-UiO-66-NH_2_) with graded pore shells by a simple hydrothermal etching method and used it as a carrier to load Ru(bpy)_2_(Mcpbpy)^2+^, and successfully prepared a nanocomposite (HH-Ru-UiO66-NH_2_) with an excellent ECL signal. The multilayer structure and cavity of HH-UIO-66-NH_2_ allowed the macromolecule Ru(bpy)_2_(Mcpbpy)^2+^ to be immobilized not only on the surface of MOF but also on the interior of MOF, which led to a significant increase in the loading of MOF on the luminescent group. On the other hand, the multilayer structure of HH-UIO-66-NH_2_ allowed the rapid diffusion of reactants, ions, and electrons, thus promoting the excitation of more luminophores. In addition, the etched HH-UIO-66-NH_2_ exposes more amino groups, which can catalyze the co-reactant K_2_S_2_O_8_ to generate SO_4_^•−^ radicals, and thus greatly improve the ECL luminophore utilization rate. Ultimately, the authors used HH-RU-UIO-66-NH_2_ as a high-performance ECL probe combined with a catalytic hairpin assembly (CHA) enzyme-free amplification technology to construct an ECL biosensor for the ultrasensitive detection of TB. The successfully prepared ECL immunosensor exhibited a good linearity in the range of 100 fM–100 nM, and the LOD of the prepared ECL sensor was only 31.6 fM [55].

Hyaluronidase (Haase) is another general term for enzymes that oligomerize hyaluronic acid (HA). It can decrease the activity of hyaluronic acid in the body, thereby increasing the ability of fluid permeation in tissues. In recent years, Haase has been considered as a potential tumor marker. In a recent work, Lin et al. designed a slow-release system based on a hydrogel constructed of HA with polyethyleneimine (PEI) and a large amount of Ru(bpy)_3_^2+^-doped silica nanoparticles (Ru@SiO_2_NPs) stably dispersed in the hydrogel as an ECL signaling probe. When Haase is present, the hydrogel is decomposed by Haase, allowing Ru@SiO_2_NPs to escape from the hydrogel into the supernatant, and the concentration of Haase can be quantified by the ECL signal generated from the supernatant. Compared with previous work, this biosensor does not require large amounts of HA to immobilize the signal probe or tedious centrifugation methods to reduce background interference, and thus has an excellent sensitivity and selectivity [36].

### 3.4. Biosensors for Detecting Nucleic Acids

MicroRNAs are small single-stranded non-coding RNAs of about 19–23 nucleotides. Although the first microRNAs were discovered as early as 1993, only in recent years has the diversity and breadth of this class of genes been revealed. It is hypothesized that vertebrate genomes have up to 1000 different miRNAs, regulating at least 30% of gene expression. Moreover, microRNAs are also considered to be reliable biomarkers for various cancers and genetic diseases. With the continuous development of biotechnology, scientists have combined DNA amplification strategies, such as target cyclic amplification (TRC), catalytic hairpin assembly (CHA), and strand displacement amplification (SDA). With the ECL technique to prepare a number of efficient and sensitive ECL biosensors for the detection of nucleic acids [95,96]. As shown in Table 5, an increasing number of ECL biosensors based on porous nanocomposites have been successfully developed and applied for the sensitive detection of nucleic acids in recent years.

Recently, Yuan et al. found that when polycyclic aromatic hydrocarbons (PAHs) were used as ligands for the synthesis of MOFs, the aggregation-induced burst (ACQ) effect of PAHs could be effectively eliminated by coordination immobilization of the ligands as a way to improve the strength and efficiency of ECL. Based on this principle, a MOF (Zn-PTC) with AI-ECL properties was prepared. The molecular spacing of PTC was effectively increased by ligand immobilization in the MOF, thus eliminating the ACQ effect and resulting in a greatly enhanced ECL response of Zn-PTC. In addition, the PTCs in Zn-PTC stacked in an edge-to-edge manner to form J-type aggregates, which also promoted the enhanced ECL response. Based on the good ECL performance, an ECL biosensor for the sensitive detection of microRNA-21 was constructed by using Zn-PTC as an ECL signaling probe in combination with a dual amplification strategy of a nucleic acid exonuclease III-stimulated targeting cycle and DNAzyme-assisted cycling. The ECL sensor was able to detect microRNA-21 in the range of 100 aM to 100 pM with an efficient and sensitive LOD of 29.5 aM [68].

Additionally, Yuan’s team prepared a Py-sp2 carbon-conjugated nanosheet (Py-sp2c-CON) with ECL properties based on the condensation reaction of tetrakis(4-formylphenyl)pyrene (TFPPy) and 2,2′-(1,4-phenylene)diacetonitrile. The porous ultra-thin structure of Py-sp2c-CON can effectively shorten the material transport distance and energy transfer process of electrons, ions, and co-reactants (S_2_O_8_^2−^), which greatly enhances the ECL response of luminescent substances. Based on these advantages, an ECL biosensor for microRNA-21 detection was prepared using the Py-sp2c-CON/S_2_O_8_^2−^/Bu_4_NPF_6_ system, which has a wide linear response (100 am~1 nM) and a lower LOD (46 am) [97].

In another study, a highly efficient and sensitive ECL biosensor for the detection of miRNA-21 was constructed using a Co-MOF-ABE/Ti_3_C_2_Tx composite as an ECL luminescent substance combined with a DSN-assisted target recovery signal amplification strategy. Co-MOF has a large specific surface area and thus can be loaded with abundant luminophores. Not only that, Co-MOF also exhibits excellent catalytic properties, and the ECL response of the composite is greatly improved by these factors. The successfully prepared ECL immunosensor achieved sensitive detection of miRNA-21 in the range of 0.00001 and 10 nM with a LOD of only 3.7 fM [98].

## 4. Conclusions and Outlooks

In one word, we give a comprehensive overview of the recent progress in the construction of ECL biosensors based on porous nanomaterials. Due to their large specific surface area, high porosity, large number of active sites, adjustable structure, easy modification, and good biocompatibility, porous nanomaterials, such as OMS, MOFs, COFs, and MPFs, have a large number of applications in the field of ECL biosensors. The preparation of different types of porous nanomaterials with ECL properties were summarized and their applications in the detection of heavy metal ions, small molecules, proteins, and nucleic acids. In conjunction with the representative articles, we summarize the advantages of porous nanomaterials in the field of ECL biosensors.

Firstly, porous nanomaterials with a large specific surface area and a variety of different functional groups can provide a good modification basis for loading ECL luminescent substances, which is beneficial for the construction of high-performance ECL biosensors.

Secondly, the high porosity and adjustable pore size can provide the basis for the preparation of composites by means of various synthesis methods, such as in situ growth and encapsulation, and it can additionally provide good channels for energy transfer and substance transport such as ions, electrons, and co-reactants.

Thirdly, the loading of co-reactants and luminescent substances in an all-in-one structure or the loading of two luminescent substances with different wavelengths in the same structure based on the RET effect can shorten the substance and charge transfer paths between the luminescent substances and basis, and thus can realize the self-enhancement of the ECL response, which reflects the superiority of the synergistic effect.

Although greater progress has been made in ECL biosensors based on porous nanomaterials, there are still some problems to be paid attention to that may limit the application of porous nanomaterials in this field. One of the main issues is that most porous nanomaterials with ECL properties in the field of macromolecular sensing still suffer from a high excitation voltage and a low luminescence efficiency because of their high impedance. Therefore, the development of luminescent substances with a high conductivity, a low voltage excitation, and a high ECL efficiency are also discussed in detail. Although porous nanomaterials have been widely used in the field of ECL biosensors, the limitations of synthesis methods and synthetic materials prevent the application of organic composite materials with a poor electrical conductivity (e.g., COFs materials) from being widely used. It is worth noting that the application of MPFs and AI-ECL materials are still in the initial stage, and so is the design of COFs materials with a high electrical conductivity. Based on this stage, the design of COFs with a high conductivity and the construction of ECL biosensors based on MPFs open new directions in the field of ECL.

1. AI-ECL materials or techniques are still a new research direction in the field of sensors. The types of ligands currently used are relatively single, and the AI-ECL mechanism of most materials is almost the same. Therefore, the search for new organic ligands, the study of a new AI-ECL material reaction mechanism, and the design of synthesizing innovative structures of AI-ECL materials will be hot directions.
2. Most of the current porous nanomaterials with functional groups have poor electrical conductivities, and the functional group types are relatively single. How to prepare porous nanomaterials with a high conductivity and multiple functional groups and how to combine them with luminescent substances with different functional groups to achieve the synergistic effect between each group are still needed to pay more attention to.
3. At present, most of the ECL biosensors based on porous nanomaterials are still in the laboratory stage. The instrumentation and experimental conditions required for testing experiments are relatively strict. Consequently, combining ECL sensors with microfluidics and smartphone detection to build portable devices and instruments for environmental detection remains a great challenge.

## Figures and Tables

**Table 1:** Different synthetic strategies for OMS-based nanocomposites with ECL properties.

Nanocomposites	Methods	Luminous Body	Duration	Ref.
mSiO_2_@CdTe@SiO_2_ NSs	In situ synthesis	CdTe QDs	Microemulsion method	[30]
g-C_3_N_4_@ms-SiO_2_	Post-synthesis modification	g-C_3_N_4_	Agitating	[31]
Ru-QDs@SiO_2_	In situ synthesis	CN QDs, Ru(bpy)_3_^2+^	Microemulsion method	[32]
Ru@SiO_2_	In situ synthesis	Ru(bpy)_3_^2+^	Self-assembly	[33]
CdTe@SiO_2_	In situ synthesis	CdTe QDs	Microemulsion method	[34]
NH_2_–Ru@SiO_2_-NGQDs	Post-synthesis modification	CNQDs, Ru(bpy)_3_^2+^	Agitating	[35]
Ru@SiO_2_ NPs	In situ synthesis	Ru(bpy)_3_^2+^	Microemulsion method	[36]
SiO_2_@Ir	In situ synthesis	Ir(ppy)_3_^2+^	Microemulsion method	[37]
SiO_2_@CQDs/AuNPs/MPBA	Post-synthesis modification	C QDs	Agitating	[38]
Ru@SiO_2_	Post-synthesis modification	Ru(bpy)_3_^2+^	Agitating	[39]
SiO_2_@Ru-NGQDs	In situ synthesis	Ru(bpy)_3_^2+^	Microemulsion method	[40]

**Table 2:** Summary of different synthetic strategies for MOF composites with ECL properties.

MOF Composites	Ligands	Metal Source	Ref.
**In situ synthesis**			
MIL-101(Al)–NH_2_	NH_2_-BDC	AlCl_3_	[46]
IRMOF-3	NH_2_-BDC	Zn(NO_3_)_2_	[47]
Ru(bpy)_3_^2+^/NH_2_-UiO-66	NH_2_-BDC	ZrCl_4_	[48]
Fe(III)-MIL-88B-NH_2_	NH_2_-BDC	FeCl_3_	[49]
UiO-67	BPDC	ZrCl_4_	[50]
GSH-Au NCS@ZIF-8	2-MI	Zn(NO_3_)_2_	[51]
Zinc Oxalate MOFs	Oxalic acid	Zn(NO_3_)_2_	[52]
**Post-synthesis modifications**			
Ru-MOF-5 NFs	PTA	Zn(NO_3_)_2_	[53]
Cu/Co-MOF	2-MI	Co(NO_3_)_2_, Cu(NO_3_)_2_	[54]
HH-Ru-UiO66-NH_2_	NH_2_-BDC	ZrCl_4_	[55]
Co-Ni/MOF	2-MI	Co(NO_3_)_2_, Ni (NO_3_)_2_	[56]
AgNPs@Ru-MOF	NH_2_-BDC	ZrCl_4_	[57]
g-C_3_N_4_@NH_2_-MIL-101	NH_2_-BDC	FeCl_3_·6H_2_O	[58]
Zn-Bp-MOFs	H3BTC,4,4-dipyridyl	Zn(NO_3_)_2_	[59]
Ru-PCN-777	H_3_TATB	ZrOCl_2_	[60]
**Self-luminous MOFs**			
Eu-MOFs	5-bop	EuCl_3_	[61]
RuMOF NS	[Ru(H_2_dcbpy)_3_]Cl_2_	Zn(NO_3_)_2_	[62]
Eu-MOF	[Ru(H_2_dcbpy)_3_]Cl_2_	Eu(NO_3_)_3_	[63]
Zr-TCBPE-MOF	H_4_TCBPE	ZrCl_4_	[64]
Hf-TCBPE	H_4_TCBPE	HfCl_4_	[65]
Zr_12_-adb	H_2_adb	ZrCl_4_	[66]
Tb-Cu-PA MOF	IPA	TbCl_3_, Cu(NO_3_)_2_	[67]
Zn-PTC	PTC	Zn(CH_3_COO)_2_	[68]
Ru@Zr_12_-BPDC	BPDC, H_2_dcbpy	ZrCl_4_	[69]
Cu:Tb-MOF	IPA	TbCl_3_, Cu(NO_3_)_2_	[70]
UMV-Ce-MOF	H_3_BTC	Ce(NO_3_)_3_	[71]
PTP/Eu MOF	H_3_BTC	Eu(NO_3_)_3_	[72]
Ce-TCPP-LMOF	TCPP	Ce(NO_3_)_3_	[73]
Zn-MOF	Hcptpy	ZnSO_4_	[74]

**Table 3:** Summary of the construction of ECL biosensors based on porous nanocomposites for small molecule detection.

Analytes	Nanocomposites	Linear Range	LOD	Ref.
DES	Ru@SiO2	4.8 × 10^−4^~36.0 nM	0.025 pM	[39]
DES	UiO-67	0.01 pg/mL~50 ng/mL	3.27 fg/mL	[50]
Rutin	GSH-Au NCS@ZIF-8	0.05~100 μM	10 nM	[51]
Acetamiprid	Cu/Co-MOF	0.1 μM~0.1 pM	0.018 pM	[54]
CAP	Co-Ni/MOF	1.0 × 10^−13^~1.0 × 10^−6^ M	2.9 × 10^−14^ M	[56]
ATX-a	AgNPs@Ru-MOF	0.001~1 mg/mL	0.00034 mg/mL	[57]
Trenbolone	Eu-MOF	5 fg/mL~100 ng/mL	4.83 fg/mL	[63]
IMI	UMV-Ce-MOF	2–120 nM	0.34 nM	[71]
Lincomycin	PTP/Eu MOF	0.1 mg/mL~0.1 ng/mL	0.026 ng/mL	[72]

**Table 4:** Summary of the construction of ECL biosensors based on porous nanocomposites for protein detection.

Analytes	Nanocomposites	Linear Range	LOD	Ref.
HE4	g-C_3_N_4_@ms-SiO_2_	10^−5^ to 10 ng/mL	3.3 × 10^−6^ ng/mL	[31]
PSA	Ru@SiO_2_	10^−15^ to 10^−6^ g/mL	0.169 fg/mL	[33]
AFP	CdTe@SiO_2_	1.0 pg/mL to 100 ng/mL	0.22 pg/mL	[34]
HAase	Ru@SiO_2_ NPs	2 to 60 U/mL	2 U/mL	[36]
BNPT	SiO_2_@Ir	0.1 ng/mL to 200 ng/mL	0.03 ng/mL	[37]
AFP	SiO_2_@CQDs/AuNPs/MPBA	0.001 to 1000 ng/m L	0.0004 ng/mL	[38]
PCT	MIL-101(Al)–NH_2_	0.0005 ng/mL to 100 ng /mL	0.18 pg/mL	[46]
cTnI	IRMOF-3	1 fg/mL to 10 ng/mL	0.46 fg/mL	[47]
SCCA	Fe(III)-MIL-88B-NH_2_	0.0001 to 100 ng/mL	31 fg/mL	[49]
Aβ	Zinc Oxalate MOFs	100 fg/mL to 50 ng/mL	13.8 fg/mL	[52]
NSE	Ru-MOF-5 NFs	0.0001 ng/mL to 200 ng/mL	0.041 pg/mL	[53]
Thrombin	HH-Ru-UiO66-NH_2_	100 fM to 100 nM	31.6 fM	[55]
PCT	NH_2_-MIL-101	0.014 pg/mL to 40 ng/mL	3.4 fg/mL	[58]
MUC1	Zn-Bp-MOFs	1 pg/mL to 10 ng/mL	0.23 pg/mL	[59]
MUC1	Ru-PCN-777	100 fg/mL to 100 ng/mL	33.3 fg/mL	[60]
CYFRA21-1	Eu-MOFs	0.005 to 100 ng/mL	0.126 pg/mL	[61]
cTnI	RuMOFNSs	1 fg/mL to 10 ng/mL	0.48 fg/mL	[62]
NSE	Zr-TCBPE-MOF	0.0001 to 10 ng/mL	52 fg/mL	[64]
MUC1	Hf-TCBPE	1 fg/mL to 1 ng/mL	0.49 fg/mL	[65]
MUC1	Zr_12_-adb	1 fg/mL to 100 ng/mL	100 ng/mL	[66]
CYFRA21-1	Tb-Cu-PA MOF	0.01 to 100 ng/mL	2.6 pg/mL	[67]
MUC1	Ru@Zr_12_-BPDC	1 fg/mL to 10 ng/mL	0.14 fg/mL	[69]
ProGRP	Cu:Tb-MOF	1.0 pg/mL to 50 ng/mL	0.68 pg/mL	[70]

**Table 5:** Summary of the construction of ECL biosensors based on porous nanocomposites for nucleic acids detection.

Analytes	Nanocomposites	Linear Range	LOD	Ref.
miRNA-182	mSiO_2_@CdTe@SiO_2_ NSs	0.1 to 100 pM	33 fM	[30]
microRNA-21	Zn-PTC	100 aM to 100 pM	29.5 aM	[68]
miRNA-133a	Zn-MOF	50 aM to 50 fM	35.8 aM	[74]
microRNA-21	Py-sp2c-COF	100 aM to 1 nM	46 aM	[97]
microRNA-21	Co-MOF-ABEI/Ti_3_C_2_T_x_	0.00001 to 10 nM	3.7 fM	[98]
miRNA-155	RuMOFs	0.8 fM to 1.0 nM	0.3 fM	[99]

## Data Availability

Not applicable.
